# Transanal hemorrhoidal dearterialization: Lessons learned from a personal series of 200 consecutive cases and a proposal for a tailor-made procedure

**DOI:** 10.1016/j.amsu.2020.05.036

**Published:** 2020-05-29

**Authors:** Carlos Walter Sobrado, José Américo Bacchi Hora, Lucas Faraco Sobrado, Marcos Onofre Frugis, Sergio Carlos Nahas, Ivan Cecconello

**Affiliations:** a Department of Gastroenterology, Digestive and Colorectal Surgery Division, University of São Paulo School of Medicine, Av. Doutor Enéas de Carvalho Aguiar, 255, 05409-000, São Paulo, Brazil; b Department of Digestive Surgery, Hospital 9 de Julho, Rua Peixoto Gomide, 545, 01409-002, São Paulo, Brazil

**Keywords:** Hemorrhoids, Transanal hemorrhoidal dearterialization, Mucopexy, Recurrence

## Abstract

**Background:**

Transanal hemorrhoidal dearterialization (THD) is an effective treatment for hemorrhoidal disease (HD). However, the surgical technique is not standardized and the results for advanced HD are controversial. The aim of this study was to assess surgical outcomes after a long follow-up and compare total and partial mucopexy.

**Materials and methods:**

Between March 2011 and July 2014, THD was offered to patients with symptomatic prolapsed hemorrhoids (Grades II, III and IV). Dearterialization was performed with the guidance of Ultrasound Doppler and mucopexy for prolapsed piles, and regarded as total or partial (if less than 6 mucopexies). Post-operative complications, long-term results and patients’ satisfaction rates were analyzed.

**Results:**

200 consecutive patients were recruited with a mean follow-up of 43 months (range 29 - 57 months). HD distribution was GII (N = 35, 17.5%), GIII (N = 124, 62%), and GIV (N = 41, 20.5%). Postoperative complications included transient tenesmus (26,5%), pain (14%) and fecal impaction (2,5%). Recurrence rates were 0, 2,4% and 17,1% for prolapse (p < 0,01) and 2,9%, 4% and 9,8% for bleeding (p = 0,33) in grades II, III and IV, respectively. Total mucopexy resulted in more tenesmus (31,2%) than partial mucopexy (14,5%), (p < 0,01). After 12 weeks of follow-up, 85% of patients were either very satisfied or satisfied; 8,5% were dissatisfied.

**Conclusion:**

THD-mucopexy is safe with low overall recurrence. Grade IV HD is associated with more recurrence and postoperative complications. Total mucopexy is associated with more tenesmus, pain and fecal impaction. A tailor-made procedure with selective dearterialization and mucopexy may be the next step in this evolving technique.

## Highlights

• .THD is safe procedure for hemorrhoids with low overall recurrence of prolapse and bleeding.
• .THD for grade IV hemorrhoidal disease is associated with more recurrence.
• .THD with partial mucopexy achieves similar good results compared to total mucopexy.
• .THD with total mucopexy is associated with more postoperative tenesmus, pain and fecal impaction.
• .The number of dearterializations and mucopexies should be tailor-made.

## 1 Introduction

Hemorrhoidal disease (HD) remains one of the most common afflictions seen by surgeons and gastroenterologists worldwide. Knowledge of its existence as well as its treatments dates from time immemorial and several studies have already been published about its history, epidemiology, and treatment modalities, yet no consensus has been reached about its precise incidence, prevalence, and standard of care to date.

Treatment of HD is not straightforward because of the several different presentations and the frequent association with other anorectal ailments, such as: skin tags, thrombosis, fistulas, anal fissures, etc. Hemorrhoids may be internal, external, or both; single or multiple; have different sizes and forms. Therefore, a definitive conclusion about the ideal therapy for hemorrhoids is close to utopian.

Surgical treatment by pile excision has been regarded as the most effective way to eradicate symptoms and to avoid recurrence. Inasmuch as “radical” pile-resection became the gold-standard therapy for decades, its tempestuous post-operative course, their short-term and long-term sequelae, and a better understanding of hemorrhoid pathophysiology, have propelled colorectal surgeons to attempt other forms of treatment. Thus anoderm-preserving methods of the distal rectum and anal canal, such as dearterialization and mucosal pexy (or lifting) described in the past two decades have gained wide acceptance. Stapled mucosal resection and anopexy, also known as procedure for prolapse and hemorrhoids (PPH), and hemorrhoidal artery ligation (HAL) also known as transanal hemorrhoidal dearterialization (THD) comprise the most important advances in this direction [[1], [2], [3]]. THD, may be doppler-guided or not, and done with or without mucopexy [4,5].

In this study, we report our personal experience with one of such techniques, THD and mucopexy (THD-M), with a prolonged follow-up. We compare our experience and acquired learning of its pitfalls with other reported series, and we entertain ways to customize this evolving technique into a tailor-made procedure.

## 2 Materials and methods

Between March 2011 and July 2014, patients with symptomatic prolapsed hemorrhoids were recruited for THD-M at University of São Paulo's teaching hospital, *Hospital das Clínicas*, and from our private practice at *Hospital Nove de Julho*, São Paulo, Brazil. Grading was established according to Goligher's classification [6]. Data were collected prospectively and results were analyzed at the end of the follow-up. The aim of this study was to assess for early and late results after a long follow-up and to compare total and partial mucopexy.

Eligible patients were grade II HD refractory to conservative management (fiber-enriched diet, laxatives, and life-style changes, or ambulatory rubber band ligation), as well as grades III and IV. External hemorrhoids and skin tags were meticulously assessed. All patients were thoroughly interviewed with emphasis on evacuatory and hemorrhoidal symptoms, and subjected to complete proctologic examination including anoscopy.

All patients 50 years of age or greater underwent colonoscopy. Exclusion criteria included coagulation disorders, pregnancy, inflammatory bowel disease, previous anorectal surgery, rectal procidentia, anal incontinence, immunosuppression and anorectal cancer. Patients with external hemorrhoids and skin tags were also included. Patients were thoroughly instructed as to their diagnosis, therapeutic options, the proposed procedure and its potential complications, before the operation was carried out. All patients signed an informed consent out of free will before the operation. The hospital's ethics committee approved the informed consent form and the operative protocol. This manuscript has been reported in line with the STROCSS criteria [7].

THD-M was offered as first choice treatment for 262 consecutive patients who fitted the eligibility criteria. Medical provider restrictions or patient preferences reduced the final THD-M group to 200 patients (55 were eventually operated by stapled-hemorrhoidopexy, and 7 by conventional hemorrhoidectomy).

The degree of satisfaction with the procedure was assessed 12 weeks after the surgery, and classified into four levels: very satisfied, somewhat satisfied, indifferent or dissatisfied. Any referred complication was also written in the follow-up chart.

Statistical analysis was performed with the objective of making comparisons between the degrees of hemorrhoidal disease, type of mucopexy and surgical outcomes. To make these comparisons, Fisher's exact test and chi-square tests were used, with a significance level of 5% for two-tailed tests. The analyses were performed using IBM-SPSS software for Windows, version 20.0.

### 2.1 Operative technique

All THD-M procedures were performed under laryngeal mask airway control anesthesia. Intestinal cleansing or enemas were not done prior to the operation. The operative procedure was performed as previously described in detail by Ratto et al. [8].

Patients were positioned in the lithotomy position. Prophylactic antibiotic (ciprofloxacin i.v.) was administered for all patients. A THD Doppler Kit device was used (THD Slide**®** S.p.A., Correggio., Italy). Following lidocaine gel lubrication, the proctoscope was inserted through the anal canal reaching the lower rectum, about 4–5 cm from the anal verge. Six arterial trunks were almost always identified at 1, 3, 5, 7, 9, and 11 o'clock. The rectal mucosa and submucosa were then transfixed with an “X” suture (2-0 absorbable polyglycolic acid with a 5/8-inch needle) to ligate the artery. The depth of the transfixed stitches was easily and safely calibrated by using the pivot hole provided in the center of the proctoscope lumen. Each mucosal distal point (caudad) from these trunks was marked with electrocautery just above the pectinate line (10 mm above). Mucopexy was performed with the original proctoscope making multiple passages of a continuous suture through the mucosa and submucosa until the anorectal ring was reached at the point previously marked with the electrocautery (above the pectinate line). One firm cranial knot elevated and fixed the mucosal prolapse (mucosal lifting) thus completing the mucopexy. During the procedure, the anal canal mucosa was always spared from the suture. Mucopexy was only performed in patients with prolapsed hemorrhoids and it was regarded as partial (when a running suture was fewer than six) and as total when six sutures were performed. The type of mucopexy performed was chosen at the discretion of the surgeon at the time of the surgery.

Patients were discharged within 24 h, mostly after the first bowel movement and maintained on a liquid-rich and fiber enriched diet, as well as with emolients. Patients were advised to avoid intense physical exercise and fecal straining for at least 2–3 weeks post-operatively. If no passage of stools occurred within 48 h, patients were advised to use osmotic laxatives (lactulose or polyethylene glycol 3350) and, the unsuccessful cases were returned for medical re-evaluation. Analgesia control was done with ketoprofene 100 mg twice a day, dipirone 1 g every 6 h, or paracetamol 750 mg three times a day, for 5–7 days. Upon discharge patients were re-evaluated at 1, 3, and 12 weeks, as well as 6 and 12 months post-operatively, or by demand. They were examined and questioned about anal bleeding, prolapse, pain, fecal incontinence, and bowel habits.

## 3 Results

A total of 200 patients underwent THD-M procedure during the study period. There were 129 males (64.5%) and 71 females (35.5%). Mean age was 41 years (21–72 years). Main symptoms were bleeding (100%), prolapse (94.5%), pain (25%), and itching/burning (18.5%). Under Goligher's classification, patients' hemorrhoid distribution was grade II (35, 17.5%), grade III (124, 62%), and grade IV (41, 20.5%).

### 3.1 Early postoperative results

Early postoperative results where assessed during hospital stay, at the first and third postoperative week following the surgery. Mean operative time was 27 min (range: 23-50 min). All patients had a total of 6 dearterializations done 4–5 cm above the anal verge. Total mucopexy was done in 138 patients (69%), and partial mucopexy in 62 patients (31%). Associated procedures were necessary in 37 patients (18.5%): skin tag excision in 29 (14.5%), hypertrophic papilla excision in 3, resection of anal canal polyp in 3, and resection of a sebaceous cyst in 2. Hospital stay was 1 day for 191 patients (95.5%), 2 days for 6 patients (3%), and 3 days for 3 patients (1.5%). Time to full return to normal daily activity was 4–7 days (mean 5.2 days) for 187 patients (93.5%), and >7 days for 13 patients. Intraoperative complications were 1 (0.5%) rectal hematoma and 1 (0.5%) rectal bleeding, which were successfully treated by transfixing hemostatic 2-0 polyglactin sutures. There was no perioperative mortality, nor there was any 30-day mortality.

Postoperative (PO) complications included transient tenesmus in 53 patients (26.5%) in the first 7 PO days, and opioid-requiring pain in 28 (14%) patients. Early PO active bleeding requiring surgical intervention occurred in 3 patients (1 caused by mucosal ulceration, and 2 from loosening of one of the dearterialization running sutures). Both were successfully resolved by hemostatic suturing. Urinary retention in need of bladder catheterization occurred in 4 (2%) patients (patients were all male and older than 50 years). Fecal impaction occurred in 5 (2.5%) patients who were then treated by osmotic laxatives and/or enema administration. External hemorrhoidal thrombosis occurred in 7 (3.5%) patients, and only 2 of them required surgical intervention. Anal fissures in the PO period were observed in 2 patients who were successfully treated conservatively (0.5% isosorbide dinitrate ointment and laxatives) for 8 weeks.

### 3.2 Long term results

**Fig. 1:**
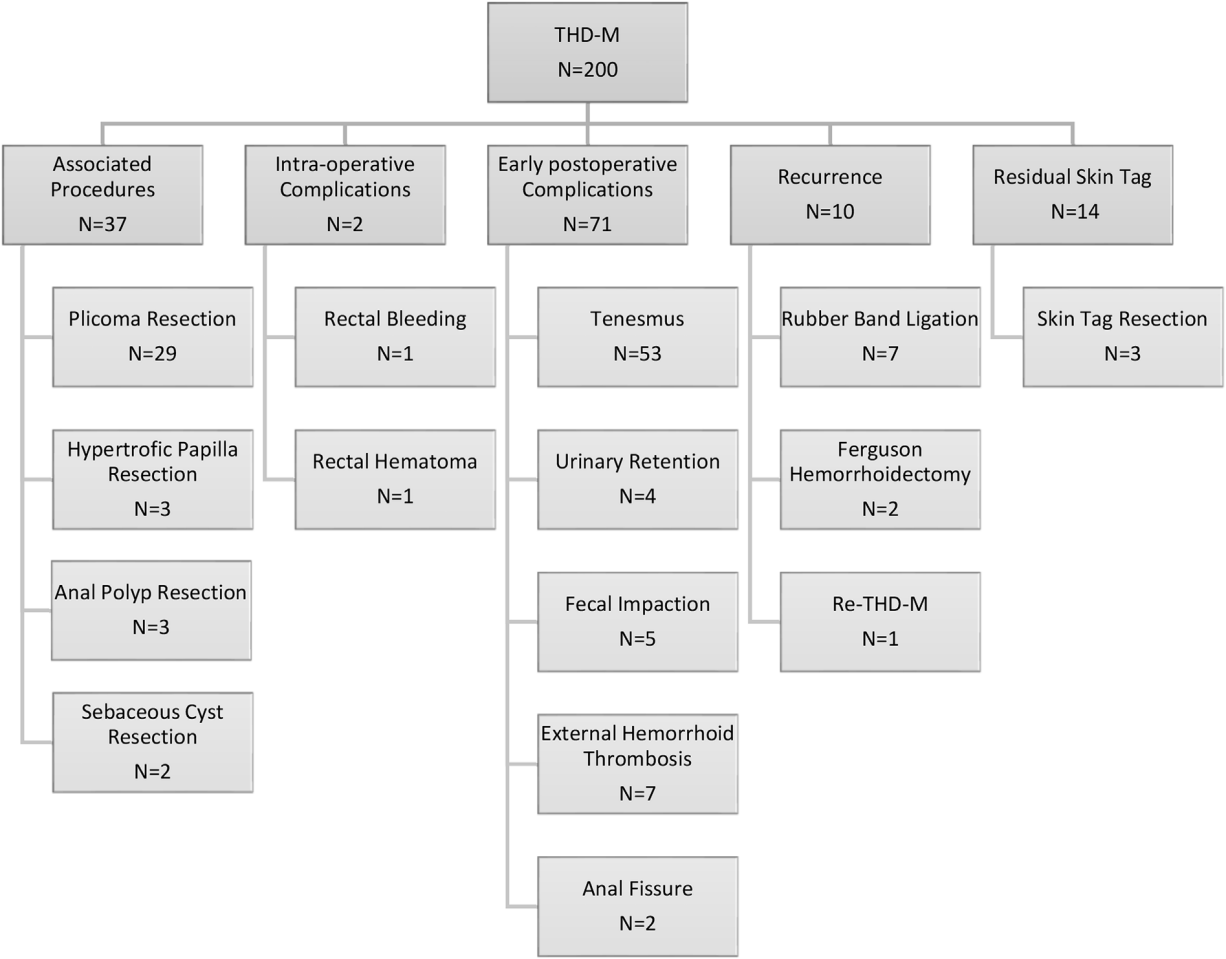
Flowchart with associated procedures, complications and recurrent disease management.

Mean follow-up time was 43 months (range 29 - 57 months). All patients were clinically re-evaluated at 12 months PO, and all were interviewed by phone at 3 months prior to the writing of this paper. At late follow-up, 10 (5%) patients presented prolapse recurrence (3 were previously classified as Grade III and 7 grade IV hemorrhoids). Seven were treated by rubber band ligation, 2 by Ferguson's hemorrhoidectomy, and 1 by re-THD-M (Fig. 1). Small recurrent anal bleeding after 1 week of PO was observed in 10 patients, all of them successfully managed by phlebotonics and suppositories. At 12 weeks PO, no patient reported bleeding. Residual skin tags were observed in 14 (7%) of patients, and excision was done in 3 patients as per patient request (pruritus and hygiene difficulty). After 12 weeks of follow-up, 67% (134/200 patients) were very satisfied, 18% (36/200) somewhat satisfied, 6,5% (13/200) indifferent and 8,5% (17/200) dissatisfied. Chronic anorectal pain or fecal incontinence were not reported at any PO time until the writing of this paper. (See Table 1)

**Table 1:** Patient characteristics.

Patients (N)	200
Median age (years-old)	41 (range: 21–72)
Gender: Female/Male	N = 71 (35.5%)/N = 129 (64.5%)
Preoperative symptoms	Bleeding (100%) Prolapse (94,5%) Itching/Burning (18,5%) Anal pain (25%)
Hemorrhoid Grades	GII N = 35 (17,5%) GIII N = 124 (62%) GIV N = 41 (20,5%)

## 4 Discussion

THD-M is a minimally invasive technique for the treatment of HD with lower rates of postoperative pain and shorter recovery when compared with conventional hemorrhoidectomy. However, there are aspects of the surgical technique that may influence outcomes and still need to be addressed, such as: the number of dearterializations, if Doppler guidance is required and if total or partial mucopexy achieve different results.

Our present study with 200 patients submitted to Doppler-guided THD, aimed to compare early and late results in regard to control of prolapse and bleeding, and also to assess if the type of mucopexy (total or partial) influences outcomes. Our mean follow-up was 43 months, which is longer than most of the series and may allow the diagnosis of late recurrences.

The operating time ranged from 23 to 50 min and the return to regular activities occurred in average at 5,2 days. Resolution of bleeding and prolapse was achieved in 95% of the patients. At the end of 12 weeks, 85% were satisfied or very satisfied, 6,5% indifferent and 8,5% were dissatisfied. Similar results have been published [9].

Surgical complications were mostly of low complexity and there was not any perioperative mortality. Reoperation due to bleeding was required in 2 patients (1%) and successfully controlled with hemostatic suturing.

Postoperative pain, defined as those who required opioids for pain control, was present in 14% of our patients. Interestingly, pain was more severe in grade IV HD (39,1%), compared with grades III (8,06%) and II (5,7%) (p < 0,01). Rubini and Tartari also reported more pain in grade IV HD and those submitted to more than four mucopexies [10]. This is probably due to local edema and transitory ischemia caused by the suture lines at the distal rectum, often referred by patients as “burning pain sensation”.

Fecal impaction is rarely reported in the literature following THD-M procedure, possibly because it is not considered a complication. All the 5 patients who had fecal impaction had grade IV HD and were submitted to total mucopexy. Up to 7% of postoperative constipation has been reported [11]. Hemorrhoidal thrombosis was diagnosed in 7 patients (3,5%). Five patients were managed clinically but other two were treated with thrombectomy. Two patients (1%) developed anal fissure and were treated with fibers, laxatives and 0.5% isosorbide dinitrate with success. This complication is infrequent and has been reported in less than 1% of the cases [5,23]. Residual skin tag was noticed in 14 patients (7%) and surgical resection was necessary in 4 due to pruritus and difficulty with hygiene. This finding has been previously reported in 3,9 to 8,3% of patients following THD [4,12,13].

The most frequent complication was tenesmus. Patients who were submitted to total mucopexy were more likely to report this complication (31,2%) than those submitted to partial mucopexy (14,5%) (p < 0,05). Total mucopexy was not associated with better control of prolapse or bleeding (Table 3). It should be noticed that the type of mucopexy was chosen at the discretion of the surgeon at the time of the surgery and that the two groups were not randomized which could have led to bias - it is possible that more complex disease (more prolapsed piles) were preferentially treated with total mucopexy. However, our impression is that HD comprises a heterogeneous group of patients – even among the same Goligher's classification -, and randomizing them according to the type of mucopexy could not have completely solved the problem. Our data show that partial mucopexy achieves good results and total mucopexy is not necessary for all patients, especially for those with fewer prolapsed piles.

Ratto et al. in the largest series published to date, reported “pain/tenesmus” in 3,1% of his patients [14]. However, their definition of “pain/tenesmus” was patients who required painkillers for more than 5 days, which was not the same definition we used in our study. It is often difficult to differentiate pain from tenesmus in the first few days PO, so we actively inquired patients about the urge to defecate and the sense of incomplete evacuation.

In regard to long-term outcomes, there was a much higher recurrence of the prolapse in grade IV HD (17,1%), when compared with grades II (0%) and III (2,4%) (p < 0,001) (Table 2). Similarly, more patients with advanced HD reported recurrence of bleeding, 9,5% of grade IV, 4,03% of grade III and 2,85% of grade II, although not statistically significant (p = 0,33). Interestingly, as noticed by other authors recurrence after THD procedure is usually just 1 or 2 piles, which allows treatment with less invasive procedures, such as rubber band ligation [9,14,15]. In our patients, rubber band ligation was performed in 7 patients, 2 were submitted to conventional hemorrhoidectomy and in 1 Re-THD-M, all with good outcomes.

**Table 2:** Postoperative results according to the degree of HD.

	Hemorrhoidal Disease (HD) (N/%)			Total	p
	Grade II	Grade III	Grade IV		
Recurrence of the prolapse	0/35 (0)	3/124 (2,4)	7/41 (17,1)	10/200 (5)	**0,001**
Recurrence of the bleeding	1/35 (2,9)	5/124 (4)	4/41 (9,8)	10/200 (5)	0,332
Pain	2/35 (5,7)	10/124 (8,1)	16/41 (39)	28/200 (14)	**0,001**
Tenesmus	6/35 (17,1)	27/124 (21,8)	20/41 (48,8)	53/200 (26,5)	**0,001**
Fecal impaction	0/35 (0)	0/124 (0)	5/41 (12,2)	5/200 (2,5)	**0,001**

**Table 3:** Postoperative results according to the type of mucopexy.

	Mucopexy (N/%)		Total	p
	Total	Partial		
Hemorrhoidal Disease				**0,001**
Grade II	6/138 (4,3)	29/62 (46,8)	35/200 (17,5)	
Grade III	94/138 (68,1)	30/62 (48,4)	124/200 (62)	
Grade IV	38/138 (27,5)	3/62 (4,8)	41/200 (20,5)	
Recurrence of Prolapse	6/138 (4,3)	4/62 (6,5)	10/200 (5)	0,504
Recurrence of Bleeding	6/138 (4,3)	4/62 (6,5)	10/200 (5)	0,504
Pain	23/138 (16,7)	5/62 (8,1)	28/200 (14)	0,105
Tenesmus	43/138 (31,2)	9/62 (14,5)	52/200 (26)	**0,013**

In the literature, there a high variability in terms of recurrence for grade IV HD, ranging from 9% [16] to up to 50% [17]. Brazilian multicenter study with 705 patients published by our group, reported a general recurrence rate of 6,4% but 26,5% for grade IV HD [18]. Ratto et al. reported recurrence of prolapse and bleeding in 18,1% of patients with grade IV and 8,7% and 8,5% for grade III and grade II respectively [14]. Giordano et al. in an attempt to reduce the recurrence rate suggested that the desarterialization and the mucopexy should be performed using two different sutures and noticed recurrence in just 1 of 31 patients (3%) [19]. However, grade IV HD was defined as “those with constant prolapse, regardless if they were reducible or not”, which is not accurate according to Goligher's classification and may have improved the results with the inclusion of grade III HD.

Anatomic studies of the anal canal have shown an important variability in terms of the number and position of the arteries that form the hemorrhoidal plexus at the distal rectum and anal canal [20,21]. Studies of the THD technique without the guidance of Doppler probe have achieved comparable outcomes [[22], [23], [24]]. This calls into question if the real benefit of THD technique arises from the ligation of a specific artery. Most likely, changes in the microcirculation and improved venous return due to the correction of the prolapse may play a role.

Other authors have assessed the arterial blood flow of hemorrhoids with Doppler guidance after the PPH technique (Procedure for Prolapse and Hemorrhoids), which removes a strip of mucosa, submucosa and not infrequently the muscular wall of the distal rectum, but have demonstrated no changes in blood flow [25,26].

In line with the presented data, we have made adjustments to our surgical technique and currently have an ongoing study to analyze the results. Instead of performing the dearterialization in the usual 6 points of the anal canal or guided by ultrasound Doppler, we selectively perform the dearterialization and the mucopexy only above the prolapsed piles. Our impression is that minimizing the sutures achieves similar outcomes in terms of recurrence but possibly less early post-operative morbidity, such as pain, tenesmus and fecal impaction.

## 5 Conclusions

In conclusion, this study confirms that THD-M is safe, achieves good long-term outcomes and high patient-reported satisfaction rates. Overall recurrence is low, but specifically in Grade IV HD, more recurrence of the prolapse is expected and patients should be informed in advance. Total mucopexy is associated with more postoperative tenesmus, pain and fecal impaction and it is not necessary for all patients. A tailor-made procedure with selective dearterialization and mucopexy just above symptomatic piles may be the next step in this evolving technique.

## Ethical approval

Ethical approval was given for this study.

## Sources of funding

There was no funding for this research.

## Author contribution

Carlos Walter Sobrado: conceptualization, methodology, investigation, writing – review & editing.

José Américo Bacchi Hora: writing, - original draft.

Lucas Faraco Sobrado: writing, - original draft.

Marcos Onofre Frugis: writing, - original draft.

Sérgio Carlos Nahas: supervision.

Ivan Cecconello: supervision.

## Registration of research studies

1. Name of the registry: CAAE.

2. Unique Identifying number or registration ID: 05156818.2.0000.0068.

3. Hyperlink to your specific registration (must be publicly accessible and will be checked).

http://plataformabrasil.saude.gov.br/login.jsf;jsessionid=4593C0F7D9E67B14B6E1795BBF09BECF.server-plataformabrasil-srvjpdf132.

## Guarantor

Carlos Walter Sobrado accepts full responsibility for the data and decision to publish.

## Consent

Written informed consent was obtained from the patient for publication of this case report and accompanying images. A copy of the written consent is available for review by the Editor-in-Chief of this journal on request.

## Provenance and peer review

Not commissioned, externally reviewed.

## Declaration of competing interest

There are no relevant conflicts of interest or disclosures.
